# Editorial: Spotlight on the relationship between visual experience and myopia

**DOI:** 10.3389/fmed.2024.1440572

**Published:** 2024-07-12

**Authors:** Manrong Yu, Liqin Jiang, Jinhui Dai, Maria Liu

**Affiliations:** ^1^Department of Ophthalmology, Eye & ENT Hospital, Fudan University, Shanghai, China; ^2^Key Myopia Laboratory of NHC, Shanghai, China; ^3^Key Laboratory of Myopia, Chinese Academy of Medical Science, Shanghai, China; ^4^Singapore Eye Research Institute, Singapore National Eye Centre, Singapore, Singapore; ^5^Department of Ophthalmology, Zhongshan Hospital, Fudan University, Shanghai, China; ^6^School of Optometry, University of California, Berkeley, Berkeley, CA, United States

**Keywords:** myopia, orthokeratology, behavior, multifocal rigid gas-permeable lens, vergence

Myopia, or nearsightedness, stands as a prevalent ocular health issue affecting the global population, particularly impacting children and adolescents. The swift increase in myopia prevalence, especially in its high myopia form, is linked to a spectrum of factors including genetic predisposition, environmental influences, and shifts in lifestyle. As we venture into new realms of discovery and intervention, this editorial is dedicated to presenting a comprehensive overview of the latest advancements in myopia research. It emphasizes behavioral, pharmacological, and optical strategies aimed at preventing and managing the progression of myopia.

Utilizing questionnaires and vision tests, Alvarez-Peregrina et al. compared the changes of lifestyle and the effects on vision in myopic and pre-myopic children before and after the COVID-19 lockdown in Spanish children aged 5–7. Findings indicated that pre-myopic children spent more time outdoors than myopic children prior to the lockdown, with no differences observed post-lockdown. Following the lockdown, there was a noted increase in outdoor activities and a decrease in digital device usage across all groups. This finding stands in contrast to earlier studies (1, 2), attributed to the eased restrictions in Spain following October 2020. Additionally, the study revealed that children's spherical equivalent (SE) values shifted to be more positive after the lockdown, associated with increased outdoor time. The conclusion drawn suggests that the rebound in outdoor engagement post-lockdown may contribute to mitigating the progression of myopia.

Yi et al. presented a study scrutinizing the precision of employing the vergence formula for myopia screening in children. Conducted at the Beijing Tongren Hospital, this cross-sectional study involved 336 children aged 6–12 with refractive errors. Biometric measurements were recorded, and the spherical equivalent (SE) was calculated using the vergence formula, based on the axial length, corneal curvature, anterior chamber depth, refractive errors, and lens position. Following cycloplegic refraction, the subjective SE was documented. The study determined no significant discrepancy between the calculated SE and the actual SE. A robust positive correlation was identified between the vergence formula-derived SE and the actual SE, signifying that the vergence formula can be effectively utilized to assess myopia in children and adolescents with high accuracy, independent of cycloplegic refraction.

Harb et al. studied the association of the indoor and outdoor human behavior with myopia by employing objective and dynamic methodologies. The research endeavored to delineate the behavioral patterns and environmental lighting conditions of young university students, both myopic and non-myopic. Participants were equipped with an Actiwatch for a continuous 3-week period to log activity and light exposure. The study discovered variations in the timing of outdoor activities, with myopic individuals tending to engage in outdoor activities later in the day, particularly on weekends. Although a trend suggesting a link between higher outdoor light levels and shorter axial lengths was observed, no significant correlation with myopia status was established. The study also highlighted that participants generally overestimated their time spent outdoors relative to Actiwatch-estimated metrics. The research concludes that while no substantial myopia-related behavioral differences were identified, the implementation of wearable technology offers an enhanced, objective methodology for behavior quantification in future myopia studies.

Queirós et al. assessed the efficacy of the Orthokeratology Double Reservoir Lens (DRL) vs. Single Vision Lenses (SVL) on axial elongation in myopic children over a 6- and 12-month period in France. The study included 48 patients aged 7–17 who received either orthokeratology treatment or single-vision spectacle correction, and concluded that orthokeratology achieved an 86 and 70% reduction in axial elongation after 6 and 12 months of lens wear, respectively, when compared to the single-vision spectacles group. It was noted that myopia progression was more pronounced in younger children, underscoring the significance of initiating myopia control measures at an early age.

Yu et al. conducted a study to evaluate the potential of a novel custom-designed rigid gas-permeable (RGP) contact lens in controlling high myopia by comparing it with single-vision spectacles. The retrospective review analyzed children fitted with spectacles or multifocal RGP lenses between January 2018 and May 2020. The study concluded that the rate of axial length increase was similar in both the control (spectacles) and multifocal RGP lens groups, indicating that multifocal RGP lenses did not significantly influence the control of high myopia progression compared to spectacles.

Together, these articles offer a multifaceted perspective on myopia research, reflecting the complexity of this global health challenge. They emphasize the necessity for a multidisciplinary strategy that integrates behavioral science, optical technology, pharmacology, and epidemiology. As we progress, it is crucial to encourage collaboration among researchers, clinicians, and policymakers to devise effective strategies aimed at curbing the escalating prevalence of myopia.

## Author contributions

MY: Supervision, Investigation, Funding acquisition, Conceptualization, Writing – review & editing, Writing – original draft. LJ: Writing – review & editing. JD: Writing – review & editing. ML: Writing – review & editing.
